# Elucidating the pathogenesis of bladder cancer through single-cell chromatin accessibility and DNA methylation analysis

**DOI:** 10.1016/j.gendis.2025.101578

**Published:** 2025-03-03

**Authors:** Yu Xiao, Lingao Ju, Gang Wang, Wan Jin, Hongwei Peng, Zongning Zhou, Mengxue Yu, Yi Zhang, Kaiyu Qian, Xinghuan Wang

**Affiliations:** a Department of Urology, Zhongnan Hospital of Wuhan University, Wuhan, Hubei 430071, China; b Department of Biological Repositories, Human Genetic Resources Preservation Center of Hubei Province, Zhongnan Hospital of Wuhan University, Wuhan, Hubei 430071, China; c Hubei Key Laboratory of Urological Diseases, Laboratory of Precision Medicine, Zhongnan Hospital of Wuhan University, Wuhan, Hubei 430071, China; d Euler Technology, ZGC Life Sciences Park, Beijing 102206, China; e High Performance Computing Center, The Peking-Tsinghua College of Life Sciences, Peking University, Beijing 100091, China; f Wuhan Research Center for Infectious Diseases and Cancer, Chinese Academy of Medical Sciences, Wuhan, Hubei 430071, China; g Medical Research Institute, Frontier Science Center for Immunology and Metabolism, Taikang Center for Life and Medical Sciences, Wuhan University, Wuhan, Hubei 430071, China

Previous studies have sought to classify bladder cancer (BLCA) into different molecular subtypes to understand its pathogenic pathways and uncover specific treatments.^1^ These subtypes, often based on genetic, transcriptomic, or proteomic profiles, aim to stratify patients for precision medicine and improve therapeutic outcomes. Despite these efforts, such classifications have rarely been applied in clinical practice due to challenges in standardization, reproducibility, and limited translational studies validating their utility.^1^ The treatment of BLCA predominantly relies on surgery, often combined with chemotherapy, immunotherapy, targeted therapy, or antibody-drug conjugates. Radical cystectomy remains the cornerstone for muscle-invasive bladder cancer (MIBC), while transurethral resection and intravesical therapy are common for non-muscle-invasive bladder cancer (NMIBC).^2^ However, the choice of its treatment modality still depends specifically on whether the disease is NMIBC or MIBC, rather than on the various molecular subtype classifications.^3^ Bridging the gap between molecular research and clinical application remains a significant challenge, highlighting the need for robust biomarker validation and the development of treatment algorithms that incorporate these subtypes to better guide personalized therapy.

Recent advancements in single-cell analytics have revolutionized our understanding of the complex molecular mechanisms underlying MIBC. Through our recent integrative analysis via genomic technologies, our research provides groundbreaking insights into the epigenetic and transcriptional landscape of BLCA, with a particular focus on transmembrane-4-L-six-family-1 (TM4SF1)-positive cancer subpopulations (TPCS).^4^ Our work utilized a novel tool named EpiTrace, developed by our group,^5^ to track single-cell evolution via chromatin accessibility. This approach allowed us to reveal the intricate mechanisms of intra-tumor heterogeneity across all stages of BLCA.^4^ Moreover, our previous study found that TM4SF1 was up-regulated in human MIBC tissues, and associated with T stage, lymph node metastasis status, and survival rate of MIBC patients.^4^

The current study, a commentary analysis of our recent TPCS paper,^4^ delves into the chromatin accessibility and DNA methylation patterns at differentially methylated regions (DMRs) to unravel the pathogenesis and progression of BLCA, with a particular focus on the transition from non-muscle invasive to muscle-invasive subtypes.

By analyzing single-cell chromatin accessibility data, single cells were classified into various clusters: stromal (Cluster 2/3/1/4), normal epithelial (Cluster 14), and cancer (Cluster 5–13 and 15) as Figure 1A. Significant differences emerged in the behavior of hypermethylated DMRs (hyperDMRs) and hypomethylated DMRs (hypoDMRs) across these cell types within the bladder urothelium. Furthermore, unsupervised hierarchical clustering of hyperDMR levels pinpointed a small subset of core indicator DMRs that effectively represent the full DMR dataset and distinguish samples. These DMRs were categorized into four groups, strongly associated with cancer invasiveness and clinical stages.^4^ All Ta (luminal) subtype tumors showed hypermethylation at group 1 DMRs, which includes genes like GPX4, PRCKZ, and CD44.^4^ Additionally, a subgroup of T3/T4 MIBC samples^4^ demonstrated hypermethylation across all indicator DMRs, suggesting a potential origin from different cellular ancestries compared with less invasive stages. Moreover, hyperDMRs were mostly open in stromal cells yet remained closed in all epithelial cells, indicating that DNA methylation levels on most hyperDMRs are stable as BLCA cells evolve from their progenitors (Fig. 1B). Conversely, hypoDMRs showed closed chromatin in both stromal and normal epithelial cells, with varying accessibility across different cancer cell clusters (Fig. 1C). These observations suggest a complex regulatory landscape in BLCA where chromatin accessibility and DNA methylation are intricately linked, influencing gene expression and cellular phenotype.

**Figure 1:**
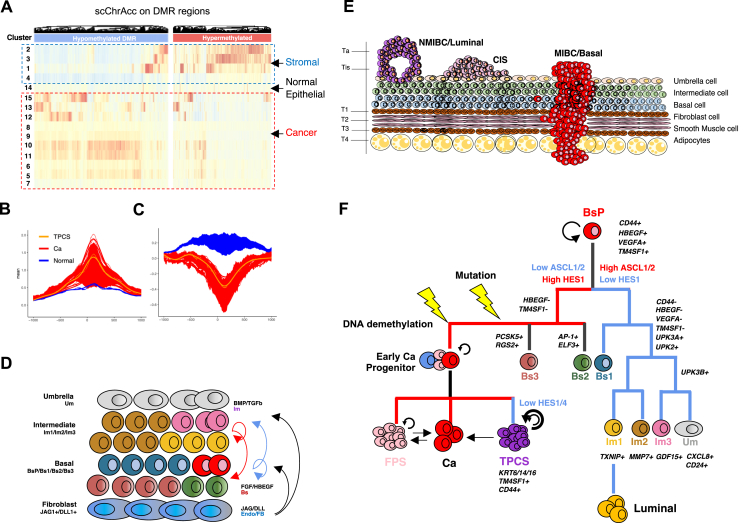
Origin of muscle-invasive and TM4SF1-positive bladder cancer (BLCA) cells in the urothelium. **(A)** Single-cell chromatin accessibility on MIBC-1685 specific differentially methylated regions (DMRs). DMRs are classified as hypomethylated or hypermethylated in BLCA. Single cells are classified as stromal (Cluster 2/3/1/4), normal epithelial (Cluster 14), or cancer (Cluster 5–13 and 15). **(B)** Muscle-invasive bladder cancer (MIBC) hypermethylated DMR (hyperDMR) chromatin was only open in stromal cells and their chromatin accessibility did not change between normal epithelial cells and cancer. **(C)** In contrast, MIBC hypomethylated DMR (hypoDMR) chromatin was closed in not only stromal but also normal epithelial cells, and their chromatin accessibility varied between different cancer cells. **(D)** Anatomy of urothelium from the basal (bottom) side to the luminal (top) side, showing cell types as well as the identified cell–cell signaling events between them. Basal and intermediate cell lineage are likely to be separated by Notch signaling activity strength, as basal cells are more proximal to the major Notch ligand provider. **(E)** The schematics summarizing bladder cell types, the clinical stages, and subtypes of BLCA. **(F)** The diagram of the evolution of BLCA in our study revealing a step-wise pathway for the emergence of the transcriptionally plastic, clinically important TM4SF1-positive cancer cell subpopulation (TPCS).

Analysis of cell–cell signaling within the bladder urothelium highlighted the roles of EGF, TGFβ/BMP, and Notch signaling. Bs1 cells uniquely provided EGF signals, whereas only intermediate cells delivered TGFβ and BMP signals to other cells.^4^ Mutual Notch signaling between basal and superficial lineage cells suggested that the physical proximity of basal cells to endothelial and fibroblast cells, which supply JAG1/DLL1 ligands (Fig. 1D), could determine cell fate and lineage bifurcation. This mutual signaling suggests that the microenvironment and intercellular communication are critical in determining cell fate within the bladder epithelium, influencing both normal and cancerous pathways.

The results of this study suggest distinct origins for different BLCA subtypes (Fig. 1E). MIBC likely originates from basal cells due to their proximity to cells providing essential Notch signals. NMIBC appears to arise from superficial intermediate or umbrella cells. These findings are summarized in detailed schematics that outline the bladder cell types, clinical stages, and subtypes of BLCA (Fig. 1F). Notably, the study charts a step-wise evolutionary pathway leading to the emergence of a transcriptionally plastic, clinically significant TPCS, which may be key to understanding the progression and treatment resistance in MIBC.

Taken together, this commentary analysis of our recently published paper regarding EpiTrace^5^ and TPCS^4^ indicates that BLCA subtypes may originate from different cells within the urothelium, driven by intricate patterns of chromatin accessibility and cell–cell signaling. The findings not only improve our understanding of the molecular biology of BLCA but also open avenues for targeted therapeutic strategies based on the molecular signatures of different cancer stages and types. This study sets the stage for future research focused on the interplay between chromatin state, DNA methylation, and tumor microenvironment in cancer progression and treatment resistance.

## CRediT authorship contribution statement

**Yu Xiao:** Writing – original draft, Methodology, Investigation, Conceptualization. **Lingao Ju:** Writing – original draft, Resources, Investigation. **Gang Wang:** Writing – original draft, Resources, Investigation. **Wan Jin:** Visualization, Software, Formal analysis, Data curation. **Hongwei Peng:** Resources. **Zongning Zhou:** Resources. **Mengxue Yu:** Resources. **Yi Zhang:** Writing – original draft, Software, Methodology. **Kaiyu Qian:** Writing – review & editing, Validation, Supervision, Resources, Project administration, Methodology, Investigation, Conceptualization. **Xinghuan Wang:** Writing – review & editing, Supervision, Resources, Project administration, Conceptualization.

## Ethics declaration

All research procedures were approved by the Institutional Review Board of the Zhongnan Hospital of Wuhan University (approval numbers: 2015029 and 2020102) and conducted in accordance with the Declaration of Helsinki.

## Data availability

The sequencing data of the human biospecimens used in this paper were derived from our previous study.^4^ The raw data and clinical information were uploaded to the Genome Sequence Archive for Human (http://bigd.big.ac.cn/gsa-human/) at the BIG Data Center, Beijing Institute of Genomics, Chinese Academy of Sciences (accession number: HRA001225). The raw sequencing data and clinical information are unique to an individual and require controlled access. The deposited and publicly available data are compliant with the regulations of the China Human Genetic Resources Management Office, Ministry of Science and Technology of China (approval number: 2022BAT0129). The materials and methods section is in the supplementary information file.

## Funding

This study was supported by the National Natural Science Foundation of China (No. 82273065), Fundamental Research Funds for the Central Universities (China) (No. 2042022dx0003), and Research Fund of Zhongnan Hospital of Wuhan University (Wuhan, China) (No. YYXKNLJS2024001, PTPP2024001). The funders played no role in the study design, data collection and analysis, decision to publish, or preparation of the manuscript.

## Conflict of interests

The authors have no conflict of interests.
