# Supplementary material for: Elucidating the pathogenesis of bladder cancer through single-cell chromatin accessibility and DNA methylation analysis
Source: Genes Dis. 2025 Mar 3;12(5):101578. doi: 10.1016/j.gendis.2025.101578 (PMC12127533; doi:10.1016/j.gendis.2025.101578)
Supplement: Multimedia component 1 [file mmc1.pdf]

Supplementary Information

Elucidating the Pathogenesis of Bladder Cancer through Single-Cell Chromatin Accessibility and DNA Methylation Analysis

Materials and methods: Pages 2-6

References: Page 7

Materials and methods

ATAC sequencing data preprocessing

All assay for transposase-accessible chromatin sequencing (ATAC-seq) data processing was conducted as detailed in our previous study.[1] Raw paired-end open chromatin tagmentation (ATAC) sequencing data were mapped to the human reference genome GRCh38 using Bowtie2 with parameters (-k 10 --very-sensitive -X 2000) (https://github.com/BenLangmead/bowtie2). We removed all unmapped reads, nonuniquely mapped reads, reads with low mapping quality (MAPQ < 20), and PCR duplicates. For in-house-prepared ATAC-seq data, quality control was conducted by evaluating the insertion size and transcription start site (TSS) enrichment using custom R scripts, leveraging the GenomicRanges package (https://github.com/Bioconductor/GenomicRanges) to measure the depth ratio at the promoter region (0 bp of the TSS vs. 1 kbp ± of the TSS). The data were annotated using the GRCh38 refFlat annotation from the UCSC Genome Browser. A QC-passed ATAC-seq library must exhibit a TSS enrichment of 6, have at least 20 million mapped deduplicated sequencing fragments, and meet PCB1>0.9 and PCB2>3 criteria (https://www.encodeproject.org/pipelines). Peak enrichment was determined by combining peaks identified from MACS2 callpeak (-f BAMPE)[2] (https://github.com/taoliu/MACS) and Genrich (-r -m 1 -j; for ATAC only) (https://github.com/jsh58/Genrich). Quality control of the ATAC-seq libraries, including assessments of read length, V-plots, and TSS enrichment, was performed using custom R scripts and deepTools (https://github.com/deeptools/deepTools). Reliable peaks were identified using the Irreproducible Discovery Rate (IDR) (https://www.encodeproject.org/software/idr). We converged reliable ATAC peaks from different data sets with a minimum overlap of 1 bp and extended them to the

largest width of overlapping peaks, resulting in a set of nonoverlapping, varied-width peaks across the genome that encompass all reliable open chromatin regions.

Classification of differentially methylated regions by CUT&Tag signals

Reads from the CUT&Tag sequencing library were transformed into bigWig format using deepTools.[3] A window of ±1200 bp around differentially methylated loci was used to extract the reads. The resulting coverage matrix, containing signals for H3K27me3, H3K27ac, H3K4me3, anti-FOXA1, and anti-AR, was clustered using the K-means method in deepTools.[3] These K-means clustered regions were annotated using ChIPseeker[4] with a range of -1000 bp to +500 bp around the transcription start site (TSS) as promoters. Their relative distributions in different genomic regions were visualized using pheatmap, available at pheatmap on CRAN. Regions were labeled as 'Promoters' if they were covered by H3K4me3 in any cell type, 'Enhancers' if covered by H3K27ac, and 'Repressors' if covered by H3K27me3. We did not detect any regions with bivalent promoter/enhancer features showing double positivity for H3K4me3 and H3K27me3.

Mutation profiling

The mutation profiling process begins with mapping raw sequencing data to the GRCh37 reference genome using bwa-mem5[5] under default settings. Germline mutations are identified using the Sentieon haplotyper (Sentieon-Genomics-201808.05) and annotated with VEP6[5] (version 90.1) and SnpSift7[7] (version 4.2). For paired tumor-normal samples, germline variants are filtered based on a gnomAD global frequency of less than 0.001 and a frequency less than 0.001 within an in-house database of 20,000 patients. This filtering process also includes checking for overlaps with variants

identified in different male members of the pedigree to isolate patient-specific germline mutations.

Somatic mutations in tumor samples are detected using Sentieon TNscope (Sentieon-Genomics-201808.05) for paired samples, or using NA12878 as a reference in unpaired cases, with additional mutation calling by Pisces[8] (version 5.2.9.122). Variants identified by both Sentieon TNscope and Pisces undergo further filtering.

Copy number variations (CNVs) are called using CNVkit[9;10] with default parameters. Both germline and somatic variants are analyzed for B-allele frequency (BAF), and the tumor genome is segmented based on BAF and sequencing depth data. Allelic copy numbers for each somatic variant are determined using a hypergeometric test.

Tumor content is estimated through an in-house CNV-based linear regression method, with validation provided by Hematoxylin and Eosin (H&E) staining. Filtering criteria for variants include a minimal tumor-cell-fraction of 5% for panel sequencing and 2% for whole-exome sequencing, a minimum of 10 reads for panel sequencing or 3 for whole-exome sequencing, and minimum read depths of 500 for panel sequencing and 30 for whole-exome sequencing.

Finally, the filtered mutations are annotated using vcfanno and undergo another round of filtering against the gnomAD database to ensure a global frequency of less than 0.001. This comprehensive profiling helps in precise mutation identification and characterization in cancer genomics.

Genomic region liftover

The liftover of genomic regions between UCSC GRCh38 and GRCh37 was performed using several tools. The R package easyLift (available at https://github.com/caleblareau/easyLift), the liftover executable from the UCSC Kent

Utility (accessible at https://genome.ucsc.edu/cgi-bin/hgLiftOver), and the lift-over synteny chain files from the UCSC Genome Browser were utilized for this purpose. The target genome for all transformations was consistently GRCh38.

DNA methylation data processing

Raw bisulfite-converted DNA methylation sequencing data were either obtained from the NCBI SRA or generated in-house. The data were processed using fastp (--trim-front2 20 -w 20)[11] (see fastp) for quality control and adapter trimming. The trimmed reads were then aligned to the GRCh37+decoy reference genome using BWA-Meth under standard conditions (refer to BWA-Meth). Following mapping, the data were deduplicated and sorted using both Sambamba (visit Sambamba) and Samblaster (more on Samblaster). The CpG methylation levels were extracted using the Pile-O-Meth tool from the MethylDackel toolkit (more details at MethylDackel). The conversion rate was quality controlled by ensuring a CHH methylation level of over 99%. Basic statistics such as on-target rate and on-target coverage were further assessed using bedtools (see bedtools), while duplication rate and mapping rate were analyzed using Sambamba.

Differential methylated loci and region

CpG methylation levels (beta values, defined as the ratio of reads showing cytosine over the total read coverage at each CpG site) were quantified across the genome using Pile-O-Meth. For each CpG site, beta values from sequencing data were summarized in R (version 3.6.2) using an in-house script. Differentially methylated loci (DML) were identified based on the following criteria: (1) a p-value less than 0.01 in a T-test between control and case groups (for example, NMIBC/MIBC or BC/normal comparisons); (2)

a difference in beta values greater than 0.1 between the case and control groups. Initial differentially methylated regions (DMR) candidates were established by merging DML that were within 100 bp of each other. The average beta value for each initial DMR candidate was calculated as the mean beta of all CpG sites within the DMR. This average beta was then subjected to a T-test, and regions with a p-value less than 0.01 were selected as candidate 'seed' DMRs. Methylation difference levels were then analyzed using a circular binary segmentation approach with DNAcopy[10] to compute segments of methylation differences between the case and control groups. K-means clustering was performed on the methylation beta differences of each segment using R (version 3.6.2), and clusters encompassing the candidate 'seed' DMRs were selected as true DMR candidates.

References

1. Xiao Y, Jin W, Qian K, et al. Integrative Single Cell Atlas Revealed Intratumoral Heterogeneity Generation from an Adaptive Epigenetic Cell State in Human Bladder Urothelial Carcinoma. *Adv Sci (Weinh).* 2024;11(24):e2308438.
2. Zhang Y, Liu T, Meyer CA, et al. Model-based analysis of ChIP-Seq (MACS). *Genome Biol.* 2008;9(9):R137.
3. Ramirez F, Dundar F, Diehl S, Gruning BA, Manke T. deepTools: a flexible platform for exploring deep-sequencing data. *Nucleic Acids Res.* 2014;42(Web Server issue):W187-191.
4. Yu G, Wang LG, He QY. ChIPseeker: an R/Bioconductor package for ChIP peak annotation, comparison and visualization. *Bioinformatics.* 2015;31(14):2382-2383.
5. Li H, Durbin R. Fast and accurate long-read alignment with Burrows-Wheeler transform. *Bioinformatics.* 2010;26(5):589-595.
6. McLaren W, Gil L, Hunt SE, et al. The Ensembl Variant Effect Predictor. *Genome Biol.* 2016;17(1):122.
7. Cingolani P, Patel VM, Coon M, et al. Using Drosophila melanogaster as a Model for Genotoxic Chemical Mutational Studies with a New Program, SnpSift. *Front Genet.* 2012;3:35.
8. Dunn T, Berry G, Emig-Agius D, et al. Pisces: an accurate and versatile variant caller for somatic and germline next-generation sequencing data. *Bioinformatics.* 2019;35(9):1579-1581.
9. Talevich E, Shain AH, Botton T, Bastian BC. CNVkit: Genome-Wide Copy Number Detection and Visualization from Targeted DNA Sequencing. *PLoS Comput Biol.* 2016;12(4):e1004873.
10. Venkatraman ES, Olshen AB. A faster circular binary segmentation algorithm for the analysis of array CGH data. *Bioinformatics.* 2007;23(6):657-663.
11. Chen S, Zhou Y, Chen Y, Gu J. fastp: an ultra-fast all-in-one FASTQ preprocessor. *Bioinformatics.* 2018;34(17):i884-i890.
